# *In vitro *activation and enzyme kinetic analysis of recombinant midgut serine proteases from the Dengue vector mosquito *Aedes aegypti*

**DOI:** 10.1186/1471-2091-12-43

**Published:** 2011-08-09

**Authors:** Alberto A Rascón, Johnathon Gearin, Jun Isoe, Roger L Miesfeld

**Affiliations:** 1 Department of Chemistry & Biochemistry, and Center for Insect Science West Room 518, 1041 E. Lowell St., University of Arizona, Tucson, AZ, 85721, USA; 2 Department of Chemistry & Biochemistry, BioSciences West Room 518, 1041 E. Lowell St., University of Arizona, Tucson, AZ, 85721, USA; 3 Sandler Center for Drug Discovery, Byers Hall, 1700 4th Street N509, University of California at San Francisco, San Francisco, CA. 94143, USA

**Keywords:** trypsin, Aedes aegypti, hemoglobin, serum albumin, zymogen

## Abstract

**Background:**

The major Dengue virus vector *Aedes aegypti *requires nutrients obtained from blood meal proteins to complete the gonotrophic cycle. Although bioinformatic analyses of *Ae. aegypti *midgut serine proteases have provided evolutionary insights, very little is known about the biochemical activity of these digestive enzymes.

**Results:**

We used peptide specific antibodies to show that midgut serine proteases are expressed as zymogen precursors, which are cleaved to the mature form after blood feeding. Since midgut protein levels are insufficient to purify active proteases directly from blood fed mosquitoes, we engineered recombinant proteins encoding a heterologous enterokinase cleavage site to permit generation of the bona fide mature form of four midgut serine proteases (AaET, AaLT, AaSPVI, AaSPVII) for enzyme kinetic analysis. Cleavage of the chromogenic trypsin substrate BApNA showed that AaET has a catalytic efficiency (k_cat_/K_M_) that is ~30 times higher than bovine trypsin, and ~2-3 times higher than AaSPVI and AaSPVII, however, AaLT does not cleave BApNA. To measure the enzyme activities of the mosquito midgut proteases using natural substrates, we developed a quantitative cleavage assay based on cleavage of albumin and hemoglobin proteins. These studies revealed that the recombinant AaLT enzyme was indeed catalytically active, and cleaved albumin and hemoglobin with equivalent efficiency to that of AaET, AaSPVI, and AaSPVII. Structural modeling of the AaLT and AaSPVI mature forms indicated that AaLT is most similar to serine collagenases, whereas AaSPVI appears to be a classic trypsin.

**Conclusions:**

These data show that *in vitro *activation of recombinant serine proteases containing a heterologous enterokinase cleavage site can be used to investigate enzyme kinetics and substrate cleavage properties of biologically important mosquito proteases.

## Background

Female mosquitoes must acquire a blood meal from vertebrate hosts in order to obtain the proper nutrients for completion of the gonotrophic cycle [1]. The site of blood meal digestion and nutrient absorption is localized in the midgut, which is also the entry site for blood-borne human pathogens. The blood feeding behavior of mosquitoes facilitates the transmission of many human pathogens, including Dengue and yellow fever viruses, and malarial parasites [2]. Because of the resurgence in Dengue fever in tropical and subtropical regions of the world [3,4], and the global persistence of malarial parasite infections [5], it is critical to understand biochemical processes required for blood meal metabolism as a means to develop effective vector control strategies.

Blood feeding in the Dengue mosquito, *Aedes aegypti*, induces the release of proteolytic enzymes in the mosquito midgut, which leads to the degradation of blood meal proteins into peptides and amino acids. These blood protein-derived peptides and amino acids are required for the synthesis of lipid and carbohydrate stores, and as a source of energy for egg production. The major classes of digestive enzymes in blood fed female *Ae. aegypti *mosquitoes are trypsins [6,7], chymotrypsins [8,9], aminopeptidases [8,10], and carboxypeptidases [11,12]. Based on cleavage of the trypsin substrate analog BApNA (Nα-benzoyl-D, L-arginine p-nitroanilide), we recently showed that trypsin-like activity in midgut extracts of dsRNA-injected blood fed *Ae. aegypti *mosquitoes was primarily due to two serine proteases [13]. One is the early phase trypsin, AaET, and the other, is a late phase trypsin named AaSPVI, which is a member of the 5G1 superfamily [14]. RNAi based studies using BApNA assays to analyze midgut extracts for trypsin activity from 0-48 hr post blood meal (PBM) showed that AaET is the only trypsin-like protease during the first 1-6 hr PBM, whereas the AaSPVI protease accounts for up to 75% of the BApNA cleaving activity during the late phase of digestion from 12-36 hr PBM [13]. These same experiments also showed that another midgut protease, AaSPVII, contributed significantly to overall fecundity in blood fed mosquitoes. Interestingly, RNAi knock down of AaLT expression, also known as Late Trypsin, had no effect on BApNA activity in midgut extracts, even though it reduced fecundity by ~30% as did the AaSPVI RNAi knock downs [13]. These data suggest that AaSPVI, and not AaLT, is the major late phase midgut trypsin in blood fed *Ae. aegypti*, and are consistent with bioinformatic analyses of protease encoding genes in the *Ae. aegypti *genome [15].

Trypsin belongs to the serine protease family, which includes chymotrypsin, elastase, and serine collagenase [16]. During catalysis, the active site serine becomes deprotonated and functions as a nucleophile attacking the carbonyl of the peptide substrate, leading to the formation of a tetrahedral intermediate [17]. Serine deprotonation by histidine, and stabilization of the newly protonated histidine by a nearby aspartate residue, constitutes a charge relay system consisting of the classic catalytic triad Ser-His-Asp. Structures of a large number of serine proteases have shown that they contain two six-stranded β-barrels and up to six disulfide linkages, which stabilizes the tertiary structure of these often secreted enzymes [18,19]. The substrate specificity of serine proteases is determined in part by an amino acid residue in the substrate specificity pocket. For example, trypsins contain an aspartate residue at this position and cleave the peptide bond on the carboxyl terminal side of arginine and lysine residues, which form an ionic interaction with the aspartate residue [16,19]. In contrast, serine collagenases have less well-defined substrate specificity pockets, and hence display a wider range of preferred cleavage sites in protein substrates [20].

To better understand the biochemical properties of the AaET, AaLT, AaSPVI, and AaSPVII midgut serine proteases, we engineered a heterologous enterokinase cleavage site into the propeptide region of the recombinant proteins and optimized a protein purification protocol using a denaturation/renaturation strategy. We quantitatively measured enzyme activity in the AaET, AaLT, AaSPVI, and AaSPVII proteases using the artificial BApNA substrate, as well as the natural substrates serum albumin and hemoglobin.

## Results

### Design of enterokinase cleavable forms of four midgut serine proteases

**Figure 1:**
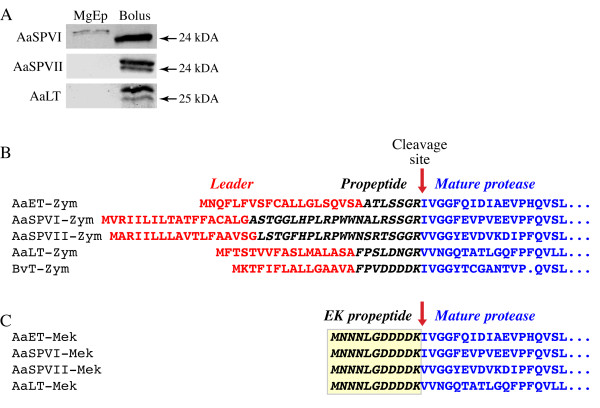
**Serine proteases in *Ae. aegypti *mosquitoes are processed to generate the mature active forms of the enzymes**. A) Western blot of midgut epithelium (MgEp) and food bolus protein extracts obtained from blood fed mosquitoes at 24 hr PBM using antibodies specific for AaSPVI, AaSPVII, and AaLT as previously described [13]. Each lane contains protein from 0.2 midgut equivalents obtained from pooled blood fed mosquitoes. Note that it is not possible to use a protein loading control for these two extracts because the amount of protein differs by >5-fold (MgEp versus food bolus), and moreover, the MgEp proteins are from mosquito origin and the food bolus proteins are from bovine origin. B) Amino acid sequence of the nascent polypeptide (zymogen) for the four mosquito serine proteases (AaET, AaSPVI, AaSPVII, AaLT) and bovine trypsin (BvT). The protein leader sequence was predicted by SignalP 3.0, whereas the propeptide sequence was defined as the sequence between the leader sequence C-terminus and the N-terminus of the mature form of the proteases based on the known amino acid sequence of the BvT mature form. The predicted propeptide cleavage site is shown. C) Amino acid sequence of the Mek forms of the four recombinant mosquito proteases showing the engineered EK propeptide sequence.

Most digestive proteases in animals are synthesized as inactive precursor proteins that are proteolytically cleaved to generate the mature enzymatically active form. To determine if this is also true of the *Ae. aegypti *AaSPVI, AaSPVII, and AaLT midgut proteases, we used peptide specific antibodies in Western blots of midgut extracts from blood fed mosquitoes. For these experiments, female *Ae. aegypti *mosquitoes were blood fed, and 18-24 hr later, midguts were removed by dissection and divided into two protein fractions, the midgut epithelium and the food bolus. As shown in Figure 1A, all three proteases were found to be present in two forms, with the smaller of the two proteins corresponding to the predicted molecular weight of the enzymatically active mature form. In the case of AaSPVI, the predicted zymogen form was primarily localized to the midgut epithelium, with only the mature form found in the food bolus, whereas both the zymogen and mature forms of AaSPVII and AaLT were found in the food bolus.

As shown in Figure 1B, we used bioinformatics to predict the most likely propeptide cleavage site in the AaSPVI, AaSPVII, and AaLT proteases based on amino acid sequence alignment, which also included the early phase trypsin, AaET, and bovine trypsinogen. Using the SignalP 3.0 algorithm to detect leader sequences in the full-length proteins [21], each of the four mosquito serine proteases was predicted to contain a leader sequence targeting the nascent polypeptide to the endoplasmic reticulum. By aligning the amino acid sequences of the four mosquito proteases with that of bovine trypsinogen using ClustalW [22], it can be seen that the most likely propeptide cleavage site is between the amino acids GR-IV (AaET, AaSPVI) or GR-VV (AaSPVII, AaLT).

We were unable to activate purified zymogen forms of these proteases *in vitro*, and high level bacterial expression and purification of recombinant mature forms of these four proteases failed to yield sufficient quantities of catalytically active enzymes (data not shown). Therefore, we engineered a heterologous enterokinase cleavage site (DDDDK) into an artificial propeptide region referred to as Mek, which permitted purification of inactive enzyme that could be activated *in vitro *using commercial preparations of enterokinase [23,24]. As shown in Figure 1C, the heterologous DDDDK enterokinase cleavage site was linked to the N-terminal sequence MNNNLG derived from pMAL-c4E, which we found to be associated with efficient Mek protein cleavage by enterokinase in an *in vitro *reaction.

### Purification of recombinant mosquito midgut serine proteases in soluble form

Based on other recombinant protease purification protocols that have been described [25-27], we optimized a protein denaturation and refolding strategy to purify soluble Mek forms of the proteases following IPTG induction. We found that the pET29a vector and BL21-DE3 bacterial expression system yielded the highest amounts of AaSPVI-Mek, AaSPVII-Mek, and AaLT-Mek proteins, whereas the AaET-Mek protein was optimally expressed using the pET28a vector. Following denaturation/renaturation, the soluble Mek proteins were cleaved *in vitro *at 4°C with recombinant enterokinase to remove the propeptide (see Figure 1C), and then further purified using either a benzamidine column for AaSPVI (cleaved AaSPVI-Mek), an ion exchange column for AaSPVII (cleaved AaSPVII-Mek) and AaLT (cleaved AaLT-Mek), or a combination of a Ni^2+^-chelating column and ion exchange column for AaET (cleaved AaET-Mek).

**Figure 2:**
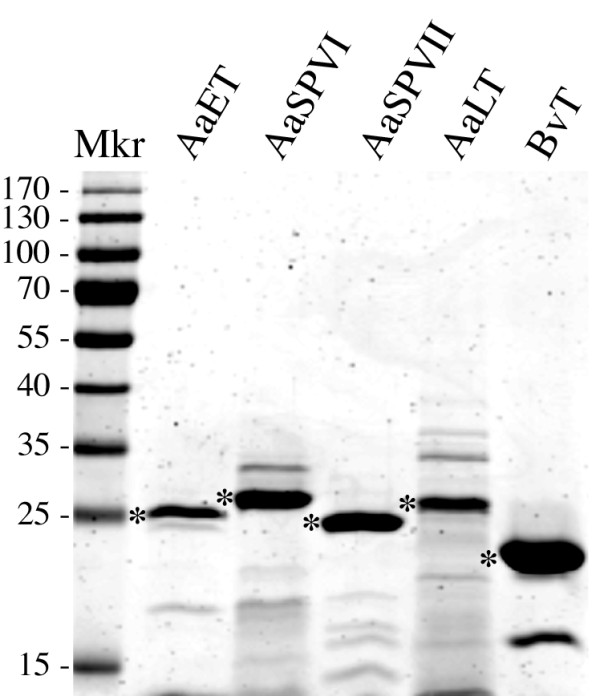
**Representative gel of purified active mature mosquito proteases and bovine trypsin used in the enzyme assays**. Each lane contains 10 μg of protein. The asterisk denotes the protein band corresponding to the expected mature protease based on Western blots of *Ae. aegypti *midgut extracts from blood fed mosquitoes [13].

As shown in Figure 2, these fractionation methods led to protein preparations containing the mature form of each mosquito protease, which were ~90% pure based on SDS-PAGE analysis. Importantly, we found that degradation of the *in vitro *activated proteases could be minimized by incubating the enterokinase cleavage step at 4°C, and including 1 mM DTT in the purification buffers (with the exception of the Ni^2+^-chelating step). The rationale for this was based on the fact that serine proteases contain multiple disulfide bonds that must be properly formed to obtain an enzymatically active protein [19]. The DTT was removed during dialysis at 4°C to promote disulfide bond formation and enzyme activation.

### Enzyme kinetic analysis of mosquito proteases using BApNA as a substrate

To determine if the enterokinase cleaved forms of the mosquito proteases were enzymatically active, we used the BApNA assay as an indicator of trypsin-like proteolytic function, and compared the BApNA cleaving activities to that of bovine trypsin (BvT). For these experiments, enzyme reaction rates were determined under steady state conditions at 24°C from linear portions of the A_405 nm _versus time plots using the extinction coefficient ∑_405 nm _= 8800 M^-1 ^cm^-1 ^[28]. The absorbance data were fit to the Michaelis-Menten equation and statistically analyzed by unpaired Student's t-test using GraphPad Prism software. One unit of enzyme activity was defined as 1.0 μmol of p-nitroaniline cleaved per min per mg of protein. The results are shown in Table 1 where it can be seen that the specific activities of the AaET and BvT preparations were comparable, whereas the specific activities of AaSPVI and AaSPVII preparations were 5-10 times lower than AaET and BvT. As predicted from recent *in vivo *RNAi studies [13,29], AaLT did not cleave BApNA under any conditions, which directly demonstrates that AaLT lacks trypsin-like activity. Also listed in Table 1 are the catalytic efficiencies of the enzymes (k_cat_/K_M_), which show that AaET is ~30 times more efficient than BvT, primarily due to a much lower K_M _for BApNA than bovine trypsin. The AaSPVI and AaSPVII mosquito proteases were also found to be more efficient in the BApNA cleavage assay than BvT, with k_cat_/K_M _values that were ~15 times higher.

**Table 1:** Steady-state kinetic parameters of mosquito proteases and bovine trypsin using BApNA as a substrate

				Specific Activity
Protease*^a^*	K_M(BApNA) _(μM)	k_cat _(s^-1^)	k_cat_/K_M _(mM^-1 ^s^-1^)	(μmol min^-1 ^(mg of protein)^-1^)
AaET	63.0 ± 1.3	2.03	32.3	5.08
				
AaSPVI	11.8 ± 0.3	0.16	13.5	0.40
				
AaSPVII	19.2 ± 1.0	0.35	18.5	0.88
				
AaLT	ND	ND	ND	ND
				
Bovine Trypsin	1051 ± 54	1.30	1.2	3.28
				

*^a^*Reaction conditions: 20 mM TRIS-HCl+10 mM CaCl_2 _pH 8.0 and 24°C.

ND: Not detected, exceeded lower level of detection for p-Nitroaniline (cleavage product of BApNA) >0.0125 abs units.

Values were determined from a mean of three determinations.

### Enzyme activity assays based on *in vitro *cleavage of albumin and hemoglobin

**Figure 3:**
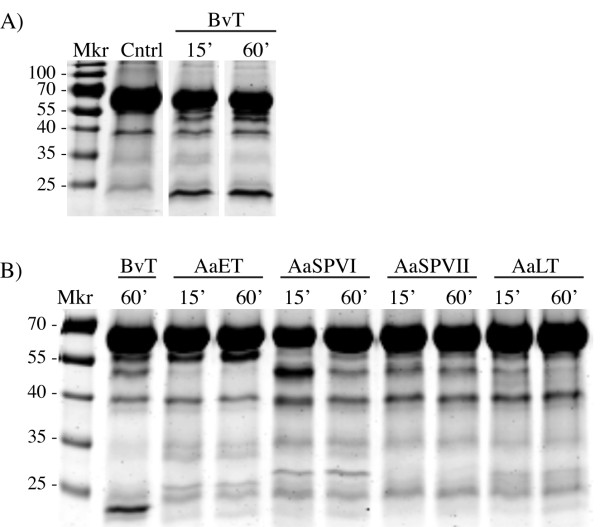
**Partial proteolysis of BSA using BvT or the activated forms of each mosquito enzyme**. A) Partial proteolysis of BSA in the absence (Cntrl) or presence of BvT for 15 or 60 min. The reactions were terminated with SDS-PAGE loading buffer and boiling for 3 min. The gel is stained with GelCode Blue reagent. B) Partial proteolysis products of BSA generated by *in vitro *cleavage reactions using each of the indicated enzymes for 15 or 60 minutes. The mass ratio of BSA:protease was 10:1 for all reactions except for the AaSPVII reaction, in which the mass ratio of BSA:protease was 298:1 owing to the high BSA cleavage activity of this enzyme relative to the others.

We developed a quantitative *in vitro *cleavage assay using bovine serum albumin (BSA) and hemoglobin (Hb) proteins to determine if the relative cleaving activities of the recombinant enzymes using BApNA were comparable to results using BSA and Hb as *in vitro *substrates. As shown in Figure 3, partial proteolysis assays using BSA demonstrated that all four recombinant mosquito proteases degrade BSA to the same extent as BvT under these conditions, including AaLT. We did however, notice some differences in the BSA cleavage patterns between the enzymes, which could be indicative of preferred cleavage sites.

**Figure 4:**
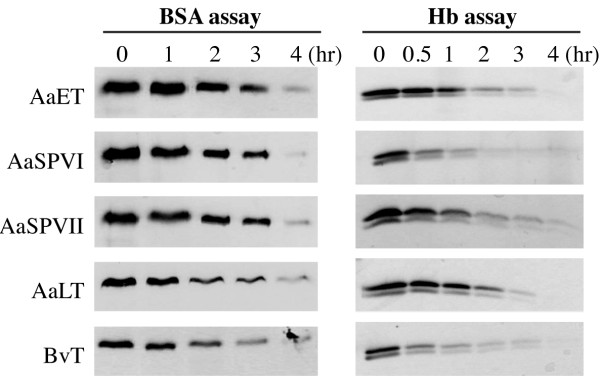
**Representative gels of albumin (BSA) and hemoglobin (Hb) digestion by activated proteases for use in quantitating enzyme activities using natural blood protein substrates**. The doublet in the Hb digestion reactions corresponds to the α and β subunits, which were both present in the commercial Hb preparation. For these experiments, all five enzymes were used at the same mass ratios, which were 922:1 for BSA:protease and 214:1 for Hb:protease. Rates of digestion from similar digestion reactions were determined using image analysis quantitation as described in Materials and Methods. Digestion data from multiple reactions run in triplicate are presented in Table 2.

To quantitatively compare the proteolytic activities of the mosquito proteases to each other, and to BvT, we measured disappearance of the full-length protein substrates, BSA (72 kDa) and Hb (15 kDa), using conditions that led to complete digestion of the protein substrate over a 4 hr incubation period. Representative examples of these cleavage assays are shown in Figure 4, with the analyzed data set presented in Table 2. For these calculations, protease activity was determined by measuring the digestion rate (loss of intact substrate over time), and dividing it by the amount of enzyme used in the assay. It can be seen in Table 2 that all four mosquito proteases and BvT had similar enzyme activities using BSA as the substrate, however, when using Hb as the protein substrate, AaSPVI was found to be 3.4 times more active than AaET, AaSPVII, and AaLT. In contrast, AaSPVI was found to have a lower catalytic efficiency in the BApNA assay (Table 1) than either the AaET or AaSPVII enzymes.

**Table 2:** Protease activity analysis using the natural substrates serum albumin and hemoglobin

Protease	BSA Digestion Rate ^a^	BSA Protease Activity ^b^	Hb Digestion Rate ^a^	HB Protease Activity ^b^
AaET	0.253 ± 0.027	42	1.66 ± 0.08	55
				
AaSPVI	0.341 ± 0.008	57	5.34 ± 0.43	177
				
AaSPVII	0.247 ± 0.014	41	1.60 ± 0.08	52
				
AaLT	0.294 ± 0.003	47	1.61 ± 0.15	52
				
Bovine Trypsin	0.291 ± 0.016	49	5.53 ± 0.12	186

^a^Units of nM min^-1^

^b^Units of nmol min^-1 ^(mg protein)^-1^

Rates were determined from plots of amount of BSA or Hb (nM) over time (min).

Taken together, these BSA and Hb cleavage assays confirm recent *in vivo *functional studies using RNAi [13,29] demonstrating that all four of these major mosquito midgut proteases encode high levels of proteolytic activity against the two most abundant blood meal proteins, despite the fact that AaLT does not cleave BApNA.

### Structural homology modeling indicates AaLT is a serine collagenase

**Figure 5:**
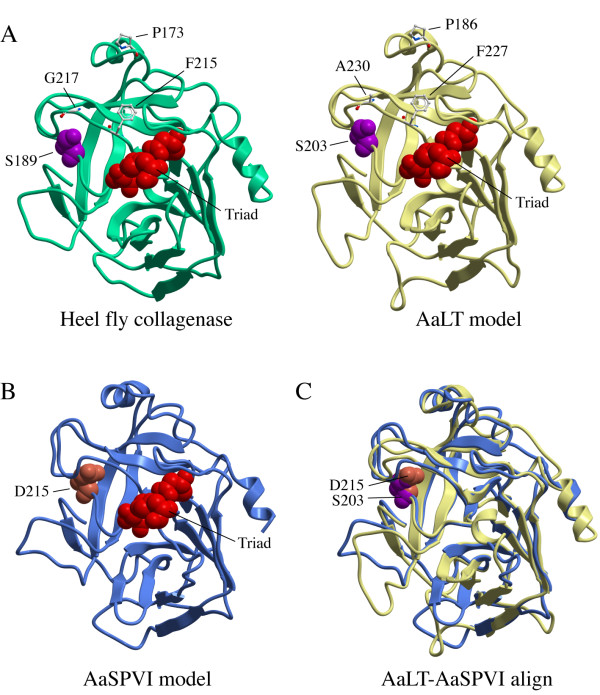
**Structural homology modeling of AaLT and AaSPVI using the Protein Model Portal (PMP) developed by the Swiss Institute of Bioinformatics**. A) Structure of the heel fly collagenase (PDB 1hyl) and AaLT model, which was generated by PMP using the heel fly collagenase structure as a scaffold. The heel fly collagenase and AaLT proteins are 40% identical at the amino acid level. Amino acid residues in the catalytic triad in the heel fly collagenase (His57, D102, S195) and AaLT model (His69, D116, S209) are shown in red CPK, along with the discerning serine residue at the bottom of the specificity pocket, which is shown in magenta CPK. Three distinct amino acids located near the extended substrate-binding site in AaLT and AaSPVI are shown in stick style and labeled. B) Structural model of AaSPVI generated by PMP using the salmon cation trypsin structure (PDB 1mbq) as the scaffold. The AaSPVI and salmon proteins are 44% identical at the amino acid level. The catalytic triad residues are shown in red CPK (His81, D125, S221), and the discerning aspartate in the specificity pocket is colored salmon CPK. C) Structural alignment of the AaLT and AaSPVI models shown in the same ribbon colors as in A and B. The S203 (AaLT) and D215 (AaSPVI) residues in the specificity pockets are shown in CPK. The structural alignment was rendered using ICM BrowserPro (Molsoft).

Since recombinant AaLT did not cleave the trypsin substrate analog BApNA (Table 1), but was just as active as the three trypsin-like proteases (Table 2), we used the protein model portal (PMP) http://www.proteinmodelportal.org to identify the most closely related structural homolog to AaLT. The PMP server aligned the input amino acid sequence with the sequences of 8.2 million comparative protein models that were generated from 3.1 million distinct UniProt entries [30]. The structural homology models for AaSPVI and AaLT were generated using 1.8Å resolution structures of salmon trypsin (PDB 1mbq), and a serine collagenase from the heel fly *Hypoderma lineatum *(PDB 1hyl), respectively. As shown in Figure 5A, the heel fly collagenase scaffold and AaLT model are nearly identical in overall structure, and both have a serine residue at the bottom of the substrate specificity pocket (S189 and S203). This is distinct from AaSPVI (Figure 5B), which has an aspartate residue in the specificity pocket (D215), as do all other trypsin enzymes [17]. An alignment of the AaLT and AaSPVI models is shown in Figure 5C, where it can be seen that in addition to amino acid differences in the specificity pocket, there are differences in the structures of several loop regions.

## Discussion

Biochemical studies of proteases are challenging for two reasons. First, most proteases are present at low amounts in tissues, and therefore must be isolated as recombinant proteins, usually from bacterial sources. However, since bacterial cells maintain a reducing environment, eukaryotic serine proteases expressed in bacterial cells are often improperly folded and localized to inclusion bodies as insoluble proteins. Second, activated proteases will autodigest if not purified under inhibitory conditions, which for structural studies of serine proteases can be done using covalent modification of the active site serine with diisopropylfluorophosphate. However, to conduct enzyme kinetic analyses, the inhibition must be reversible, or if the inactive zymogen form of the enzyme is purified, it needs to be cleaved *in vitro *using a readily available activating enzyme. As has been done in several other studies in which recombinant serine proteases were expressed in bacteria [25-27], we solved both the solubility and degradation problems. This was done by using a denaturation/renaturation protocol to refold soluble proteins, and by engineering in a heterologous cleavage site into the propeptide region to control *in vitro *activation (see Figure 1). This combined experimental approach allowed us to answer three important questions in the field of mosquito biology, 1) which kinetic parameters differentiate the four most abundant midgut serine proteases in blood fed *Ae. aegypti *mosquitoes?, 2) are the BApNA cleaving activities of these four enzymes similar to the activities determined by an *in vitro *cleaving assay using natural protein substrates, such as albumin and hemoglobin?, and 3) if the late phase AaLT serine protease is not a trypsin-like enzyme, then what is it?

Using the substrate analog BApNA, we showed that AaET is indeed a trypsin-like enzyme cleaving BApNA more efficiently (in terms of k_cat _and k_cat_/K_M_) than the three other mosquito serine proteases analyzed, including bovine trypsin (Table 1). In addition, we demonstrated that AaSPVII is also a trypsin-like enzyme, cleaving BApNA more efficiently than AaSPVI. This is an important finding because our earlier *in vivo *functional studies using an RNAi strategy, suggested that AaSPVII contributed very little to the BApNA activity in midgut protein extracts [13]. Surprisingly, AaSPVI, which was shown to be responsible for ~75% of the BApNA cleaving activity in midgut extracts in these same experiments, had the lowest specific activity in our *in vitro *BApNA assays, owing to a low turnover rate as defined by k_cat _(Table 1). It had previously been proposed that AaLT might not be a trypsin based on the presence of a serine residue, rather than an aspartate residue, in the specificity pocket [6,29]. We were able to directly test this prediction using purified active AaLT protein, and found that AaLT was indeed lacking BApNA cleaving activity, despite having similar rates of digestion to that of AaET and AaSPVI using BSA and Hb as substrates (Table 2). This result is consistent with a recent bioinformatic analysis indicating that AaLT is more closely related to serine collagenases than trypsins [14], since most serine collagenases do not cleave the substrate analog BApNA [20].

Structural homology modeling of AaLT was done using a 1.8Å serine collagenase structure from heel fly that was 40% identical to AaLT at the amino acid level (Figure 5). It can be seen from this model that a serine residue sits at the bottom of the specificity pockets of AaLT (S203) and the heel fly collagenase (S189), rather than an aspartate residue as in AaSPVI (D215) and all other bona fide trypsins [17]. It is likely that this amino acid difference in the specificity pocket of AaLT is responsible for the lack of BApNA cleaving activity. Moreover, similar to the heel fly collagenase [31], AaLT might recognize and cleave after Leu residues, rather than Arg residues, given that both enzymes contain serine residues in the specificity pocket. However, further studies are needed to test this prediction.

Why would blood fed mosquitoes secrete a collagenase-like enzyme into the midgut, considering that human blood contains very little, if any, collagen? One explanation might be that serine collagenases have been shown to be somewhat promiscuous with regard to substrate specificity, and therefore could function as garbage disposals in the digestion system, much like proteosomes do in the cytoplasm to degrade cellular proteins. This notion comes from the extensive biochemical analysis of a serine collagenase from the fiddler crab (*Uca pugilator*), which has a 37% sequence identity to AaLT. These studies showed that the crab enzyme is a true collagenase, however, it was also found to have trypsin-, chymotrypsin-, and elastase-like substrate specificities and cleavage activities owing to a substrate specificity pocket that accommodates different basic, polar, and hydrophobic amino acids [20,32,33]. For a mosquito to have such an enzyme would be advantageous, since the mosquito must digest the blood meal fairly quickly in order to obtain the proper nutrients to fuel the gonotrophic cycle.

## Conclusions

Serine proteases expressed in the midgut of blood fed *Ae. aegypti *mosquitoes are required for protein digestion and completion of the gonotrophic cycle, and could potentially be selective inhibitor targets for vector control. In order to quantitate enzyme activities in the four most abundant midgut serine proteases in *Ae. aegypti*, we engineered a heterologous enterokinase cleavage site into an artificial propeptide region to permit the purification and *in vitro *activation of recombinant enzymes. We found that three of the four proteases (AaET, AaSPVI, AaSPVII) cleave the trypsin analog BApNA in an *in vitro *assay, which confirms that they are trypsin-like serine proteases, whereas a fourth enzyme, misnamed late trypsin (AaLT), does not cleave BApNA and is structurally related to serine collagenases.

## Methods

### Chemicals and antibodies

Tris(hydroxymethyl)aminomethane (TRIS), calcium chloride, sodium chloride, guanidium hydrochloride, and DMSO were all purchased from EMD Sciences (Gibbstown, NJ), and are of reagent grade or better. Custom midgut protease polyclonal rabbit antibodies were generated by GenScript (Piscataway, NJ) and have been previously described [13]. Conjugated secondary antibodies used for Western blotting were obtained from Li-Cor (Lincoln, NE).

### Construction of bacterial expression plasmids

Total RNA from a pool of five midguts isolated from unfed mosquitoes (enriched for AaET transcripts), or blood fed mosquitoes at 24 hr PBM (enriched for AaSPVI, AaSPVII, AaLT transcripts), was isolated using TRIzol according to the manufacturer's protocol (Invitrogen, Carlsbad, CA), and converted to cDNA as previously described [13]. The genes of interest were then PCR-amplified using gene-specific primers (Table 3). Recognition sequences for NdeI and HindIII restriction sites were included in the AaET, AaSPVI, and AaSPVII primers, whereas recognition sequences for NdeI and XhoI restriction sites were included in the AaLT primer, to facilitate cDNA cloning of zymogen forms into the pET28a and pET29a vectors (Novagen, Madison, WI). A second set of protease coding sequence primers (Table 3), which include an artificial leader sequence containing an enterokinase cleavage site (DDDDK), a start codon, and an asparagine-rich propeptide region from the bacterial expression vector pMAL-c4E (New England Biolabs, Ipswich, MA), were used to construct the enterokinase site containing forms of the proteases referred to as Mek. By design, the enterokinase cleaved products of the recombinant precursor proteins were identical in amino acid sequence to the predicted mature forms of these proteases based on bioinformatics. All plasmid constructs were confirmed by DNA sequencing.

**Table 3:** Primers used for PCR amplification and cloning of recombinant forms of AaET, AaSPVI, AaSPVII, and AaLT, without (Zymogen), or with, an enterokinase cleavage site (Mek)

Protease	Primer	Primer Sequence
AaET-Zymogen	AR1-ET-ORF-F	5'-AAAAAAA**CATATG**AACCAATTTCTCTTTGTCAG-3'
	AR2-ET-ORF-R	5'-AAAAAAA**AAGCTT**ATTAAACCTCGGAAACCTCTCG-3'
		
AaSPVI-Zymogen	AR17-5G1-3714-F	5'-AAAAAAA**CATATG**GTTCGCATCATTCTTATTCT-3'
	AR18-5G1-3714-R	5'-AAAAAAA**AAGCTT**ATTACAATCCACTGACCTCCTGCACCC-3'
		
AaSPVII-Zymogen	AR15-CxLTA1-ORF-F	5'-AAAAAAA**CATATG**GCTCGTATCATCCTTCTGTT-3'
	AR16-CxLTA1-ORF-R	5'-AAAAAAA**AAGCTT**ATTAAACTCCACTGACTTCGGCC-3'
		
AaLT-Zymogen	AR4-LT-ORF-F	5'-AAAAAAG**CATATG**TTCACTTCAACGGTGGT-3'
	AR5-LT-ORF-R	5'-AAAAAAG**CTCGAG**TTATTACAGTCCAGTCTTCTGCTTGAT-3'

AaET-Mek	AR28-ET-M-pET-EK-F	5'-AAAAA**CATATG***AACAACAACCTCGGCGATGACGATGACAAG*ATCGTTGGCGGATTCCAGAT-3'
	AR2-ET-ORF-R	5'-AAAAAAA**AAGCTT**ATTAAACCTCGGAAACCTCTCG-3'
		
AaSPVI-Mek	AR27-5G1-M-pET-EK-F	5'-AAAAA**CATATG***AACAACAACCTCGGCGATGACGATGACAAG*ATTGTTGGTGGCTTTGAAGT-3'
	AR18-5G1-3714-R	5'-AAAAAAA**AAGCTT**ATTACAATCCACTGACCTCCTGCACCC-3'
		
AaSPVII-Mek	AR29-CxLT-M-pET-EK-F	5'-AAAAA**CATATG***AACAACAACCTCGGCGATGACGATGACAAG*GTCGTCGGCGGTTATGAAGT-3'
	AR16-CxLTA1-ORF-R	5'-AAAAAAA**AAGCTT**ATTAAACTCCACTGACTTCGGCC-3'
		
AaLT-Mek	AR30-LT-M-pET-EK-F	5'-AAAAA**CATATG***AACAACAACCTCGGCGATGACGATGACAAG*GTAGTAAACGGACAAACGGC-3'
	AR5-LT-ORF-R	5'-AAAAAAG**CTCGAG**TTATTACAGTCCAGTCTTCTGCTTGAT-3'

Restriction sites used for cloning of the genes of interest are shown in **BOLD**.

Primers for the mature_ek _protease constructs were designed with a rich asparagine "propeptide" region containing and enterokinase cleavage site (*ITALICIZED *and UNDERLINED).

### Bacterial expression and protein denaturation/renaturation conditions

The zymogen and Mek plasmid constructs were transformed into the Rosetta2-DE3 and BL21-DE3 bacterial expression systems (Novagen), respectively. A single colony was picked from an overnight plate grown on Luria Broth (LB) agar supplemented with 15 μg/ml kanamycin + 34 μg/ml chloramphenicol (for Rosetta2-DE3 strains) and 34 μg/ml kanamycin (for BL21-DE3 strains), and placed in 0.2 L of LB with the proper antibiotics. The overnight culture was incubated in a 37°C shaker (250 rpm) and grown overnight. From the overnight culture, 6 × 1L LB cultures with the proper antibiotics (in 2.8 L Fernbach flasks) were incubated in a 37°C shaker (200 rpm). At an OD_600 _~ 0.7-1.0, the cultures were induced with 1 mM isopropyl-β-D-thiogalactopyranoside (IPTG) and grown for 4 h. Bacterial cells were harvested by centrifugation at 6,000 rpm for 20 min (4°C). Pellets were then flash frozen in liquid N_2 _and stored at -80°C until needed.

Both the zymogen and Mek forms of the proteases were insoluble under all growth conditions tested, and therefore proteins were purified from bacterial inclusion bodies and subjected to a protein denaturation/renaturation protocol using guanidine hydrochloride (Gu-HCl). Briefly, 8 g of cell paste was lysed by sonication in buffer containing 20 mM TRIS-HCl + 10 mM CaCl_2 _+ 200 mM NaCl pH 8.0 and 1 mM DTT (lysis buffer). The cells were then centrifuged for 45 min at 16,000 rpm and 4°C. After centrifugation, the inclusion body pellet was washed by sonication (in 15 s bursts, 20% power for a total of 3 min, on ice) using lysis buffer containing 0.5 M Gu-HCl and 2% Triton X-100. The washed cells were then centrifuged at 16,000 rpm (4°C) for 15 min. The wash step was repeated twice and the pellet containing the insoluble and inactive protease was resolubilized using lysis buffer with 6 M Gu-HCl. The protein was then refolded at 4°C using dialysis (4 L 20 mM TRIS-HCl + 10 mM CaCl_2 _+ 200 mM NaCl pH 8.0) with multiple buffer changes to slowly remove the Gu-HCl and DTT. The dialyzed protein was concentrated at 4°C using an N_2 _gas pressured Amicon concentrator (with a YM-10 membrane, Millipore), aliquoted, flash frozen in liquid N_2_, and stored at -80°C. The concentrations of all purified and refolded recombinant proteins were determined using the BCA Assay kit (Pierce) with bovine serum albumin (BSA) (Pierce) as a standard.

### Activation and purification of the Mek form of recombinant proteases

To remove the artificial leader sequence of the inactive Mek proteins, each refolded protease was treated with recombinant enterokinase (rEK) obtained from New England Biolabs. All purification steps were carried out at 4°C. For AaSPVI-Mek, ~11 mg of protein was treated with ~80 units of rEK and incubated at 4°C for 24 h (reaction set in an Erlenmeyer flask with low agitation using a stir bar and plate). The reaction mixture was then purified using a HiTrap Benzamidine FF 1 ml column (GE Healthcare, Waukesha, WI) that had been equilibrated with 20 mM TRIS-HCl + 10 mM CaCl_2 _+ 200 mM NaCl pH 8.0. The column was then washed with 10 column volumes of equilibration buffer that contained 1 M NaCl and eluted with 15 column volumes of 50 mM Glycine pH 3.0. Addition of 1 M TRIS-HCl pH 8.0 to the elution fractions during purification prevented denaturation of the protein. Fractions were pooled and collected based on SDS-PAGE analysis. The protein buffer was exchanged during concentration to 20 mM TRIS-HCl + 10 mM CaCl_2 _pH 8.0.

For AaSPVII-Mek and AaLT-Mek, ~35 mg of protein was treated with 60 units of rEK and incubated at 4°C for 48 h as described above. After 48 h, the protein was diluted to obtain a final concentration of NaCl to ~16 mM, and DTT was added to 1 mM to inhibit disulfide bridge formation and minimize autodigestion. The proteins were then loaded and purified using a Q-sepharose HiTrap 5 ml column (GE Healthcare) that had been equilibrated with 20 mM TRIS-HCl + 10 mM CaCl_2 _+ 16 mM NaCl pH 8.0 and 1 mM DTT. The column was washed with 4 column volumes of equilibration buffer and eluted with a linear gradient of increasing NaCl (from 0.016 M to 1 M). Protein fractions were pooled and collected based on SDS-PAGE gel analysis. As a last step in the purification, the proteins were desalted by dialysis at 4°C in 20 mM TRIS-HCl + 10 mM CaCl_2 _pH 8.0, which helped to remove uncleaved Mek proteins owing to insolubility in low salt.

The activation and purification of AaET-Mek required using the pET28a vector (yielding a His_6 _tagged protein) in BL21-DE3 cells, as well as a modified purification protocol, owing to low protein expression levels when using the pET29a vector (yielding an untagged native protein). In the first step, ~36 mg of AaET-Mek protein was incubated with 60 units of rEK at 4°C for 24 h, and purified using a Ni^2+^-charged HiTrap Chelating HP 1 ml column according to the manufacturer's recommendation (GE Healthcare). Protein fractions were pooled and collected based on SDS-PAGE gel analysis, and the protein was buffer-exchanged by dialysis in 20 mM TRIS-HCl + 10 mM CaCl_2 _pH 8.0 buffer. The protein mixture was diluted to obtain a final concentration of 18 mM NaCl, and 1 mM DTT was added to the mixture and all purification buffers. The protein was then loaded on to a Q-seph column and purified as above. Protein fractions were pooled and collected, and dialyzed in 20 mM TRIS-HCl + 10 M CaCl_2 _pH 8.0 with 0.5 mM DTT. All active mature protein preps were concentrated using an N_2 _gas pressured Amicon concentrator (with a YM-10 membrane), aliquoted, flash frozen in liquid N_2_, and stored at -80°C. The concentrations of all activated and purified recombinant proteins were determined using the BCA Assay kit with bovine serum albumin (BSA) as a standard.

### *In vitro *BApNA Spectrophotometric Assays of Recombinant Proteases

The synthetic chromogenic substrate Nα-benzoyl-D, L-arginine-p-nitroanilide hydrochloride (BApNA) (MP Biomedicals, Solon, OH) was used to test for trypsin-like activity using 20 mM TRIS-HCl, 10 mM CaCl_2 _pH 8.0, constant enzyme concentration (20 nM (AaET), 50 nM (AaSPVI, AaSPVII, BvT), and 500 nM (AaLT) and varying substrate concentrations (steady-state conditions). Absorbance was measured as a function of time at 405 nm using the CARY WinUV Enzyme Kinetics Application on the CARY 50 UV-visible spectrophotometer (Varian Medical Systems, Palo Alto, CA). All experiments were done in triplicate and presented in Table 1 as mean values ± SEM.

### Protease assays using BSA and Hb as substrates

Partial proteolysis of BSA (Sigma Aldrich, St. Louis, MO) was performed by incubating 40 μg of BSA with active mature mosquito proteases (AaET, AaSPVI, and AaLT), or commercial bovine trypsin at a 10:1 mass ratio of BSA to protease, and for AaSPVII, at a mass ratio of 298:1 of BSA to protease. Reactions (0.1 ml) were carried out in 20 mM TRIS-HCl + 10 mM CaCl_2 _pH 8.0 and 24°C. The 8 μg protein samples (20 μl volume) were withdrawn at 15 and 60 min after the addition of protease, treated with SDS-PAGE sample buffer, and stored at -20°C until all samples collected. Once collected, samples were thawed, incubated at 95°C for 3 min, and analyzed by 12% SDS-PAGE. The SDS-PAGE gel was stained with GelCode Blue Stain Reagent (Thermo Scientific, Fair Lawn, NJ) and destained with high purity water prior to photo documenting.

Activities of the four mosquito proteases and bovine trypsin were determined using BSA and Hb as protein substrates. Reactions were initiated by incubating ~830 ng BSA with ~0.9 ng of protease and incubating ~965 ng Hb with ~4.5 ng protease in 20 mM TRIS-HCl + 10 mM CaCl_2 _pH 8.0. Reactions (0.15 ml) were carried out at 24°C and samples (15 μl) were withdrawn at t = 0, 1, 2, 3, and 4 h after the addition of protease for all BSA reactions. For AaET, AaSPVII, and AaLT Hb reactions, samples (18 μl) were withdrawn at t = 0, 0.5, 1, 2, 3, and 4 h. For AaSPVI and BT, samples (18 μl) were withdrawn at t = 0, 15, 30, 45, and 60 min. All protein samples were treated with SDS-PAGE sample buffer, which contained a final concentration of 4 mM DTT, and stored at -20°C until all samples collected. Once collected, samples were thawed, set in a 95°C water bath for 3 min, and analyzed by 12% SDS-PAGE (BSA reactions) and 15% SDS-PAGE (Hb reactions). All gels were stained prior to photo documenting using the ProteoSilver Plus Silver Stain kit (Sigma Aldrich) according to the manufacturer's protocol. NIH ImageJ software was used to calculate the pixel density of the protein bands and used to determine the amount of protein substrate (nM), which was then plotted as a function of time (min) to calculate the digestion rate (nM min^-1^). All experiments were done in triplicate and presented in Table 2 as mean values ± SEM.

### Structural homology modeling of AaLT and AaSPVI

The Protein Model Portal (PMP) http://www.proteinmodelportal.org developed by the Swiss Institute of Bioinformatics in Basel was used to identify protein structures that are most similar to the predicted structures of AaLT and AaSPVI as described by Arnold et al. [30]. The best fit for AaLT was a serine collagenase from *Hypoderma lineatum *(heel fly) that had been solved at 1.8Å resolution (PDB 1hyl), whereas the best fit for AaSPVI was a 1.8Å resolution structure of an anionic Salmon trypsin (PDB 1mbq). A structural alignment of the AaLT and AaSPVI protein models was performed using ICM BrowserPro http://www.molsoft.com software from MolSoft (La Jolla, CA), which is based on the method of Cardozo et al. [34,35].

## Abbreviations

BApNA: Nα-benzoyl-D; L-arginine p-nitroanilide; BvT: bovine trypsin; BSA: bovine serum albumin; Hb: hemoglobin; PMP: protein model portal.

## Authors' contributions

AAR, JI, and RLM designed research; AAR, JG, and JI performed research; AAR, JG, JI, and RLM analyzed data; and AAR and RLM wrote the paper. All authors have read and approved the manuscript. The authors declare no conflict of interest.
